# Self-management in condition-specific health: a systematic review of the evidence among women diagnosed with endometriosis

**DOI:** 10.1186/s12905-019-0774-6

**Published:** 2019-06-19

**Authors:** Rebecca O’Hara, Heather Rowe, Jane Fisher

**Affiliations:** School of Public Health and Preventive Medicine, Monash University, Level 4, 553 St Kilda Rd, Melbourne, Victoria 3004 Australia

**Keywords:** Self-management, Chronic disease, Endometriosis, women’s health, Self-care, Person-centred care

## Abstract

**Background:**

Endometriosis is a chronic condition, requiring long-term care as there is no cure. Self-management is the active participation of a person in managing their chronic condition and has been associated with improved knowledge, self-efficacy, performance of self-management tasks and some aspects of health status in interventions for other chronic diseases. The aim was to review the available evidence about the impact of self-management on condition-specific health among women with endometriosis.

**Methods:**

The Medline, PsycINFO, CinahlPlus, Web of Science and Scopus databases were searched and PRISMA guidelines were followed. Search terms were entered both as keywords and mapped to individual database subject headings. Inclusion criteria were: papers that reported investigations of any approach to self-management; among women (at least 18 years) diagnosed with endometriosis and published in English in a peer-reviewed journal. All study designs using quantitative or qualitative methods were eligible for inclusion. Two reviewers independently examined the quality of studies using standard criteria. The systematic review was registered with Prospero (CRD42016042028).

**Results:**

A total of 1164 records were identified (after duplicates were removed), and 27 papers, reporting 19 studies met inclusion criteria. Two papers reported findings from RCTs of complementary therapies, seven reported survey data and 18 qualitative studies. No study had investigated all elements of self-management. Women with endometriosis utilise a range of self-care activities and complementary therapies to assist them to manage their symptoms. Women reported both positive and negative experiences with health care providers.

**Conclusions:**

There is some evidence that self-care activities, complementary therapies and positive patient–healthcare provider relationships are important components of self-management for endometriosis. Self-management among women with endometriosis is an emerging field of research and no investigations of all elements of self-management, informed by a comprehensive definition and theoretical framework are available. Health and wellbeing outcomes and barriers and facilitators to self-management for women with endometriosis require further investigation.

**Electronic supplementary material:**

The online version of this article (10.1186/s12905-019-0774-6) contains supplementary material, which is available to authorized users.

## Background

Endometriosis is a chronic, relapsing, inflammatory condition characterised by endometrial-like tissue growing outside the uterus, which can result in adhesions and pain [1–3]. Endometriosis has been associated with pain with menstruation (dysmenorrhea), intercourse (dyspareunia), urination (dysuria), defecation (dyschezia), and ovulation, and lower back and chronic pelvic pain [1, 3]. Other symptoms can include heavy menstrual bleeding, gastrointestinal symptoms, subfertility or infertility, and chronic fatigue [1, 4, 5]. Laparoscopy with histological confirmation is the ‘gold standard’ for definitive diagnosis of endometriosis [3, 4].

Self-management is key to the effective management of chronic health conditions like asthma, diabetes and arthritis [6]. There is no ‘gold standard’ definition [6], but self-management is generally conceptualised as the active participation of the person in planning, decision making, and tasks to manage the symptoms, treatment, physical and psychosocial changes involved in living with a chronic condition [6, 7]. It extends beyond ‘self-care’ which is defined as the tasks an individual performs at home in order to manage symptoms of a condition [7]. Key elements of self-management are summarised in Table 1. Barlow and colleagues [6] found that compared to usual care, self-management interventions resulted in improved knowledge, self-efficacy, the performance of self-management tasks and some aspects of health status [6].

**Table 1:** Key elements of self-management [7–10]

• Active participation in decision making, treatment and management	
• Self-care tasks/behaviour change	
• Informed decision making	
• Psychosocial, emotional or social adjustments	
• Monitoring symptoms	
• Communication	
• Problem-solving	
• Patient-provider partnership	
• Self-efficacy	
• Knowledge of the condition (health literacy)/information seeking	
• Resource utilisation	

Grey and colleagues [11] developed an evidence informed conceptual framework about self- and family management of chronic conditions. It articulates the processes required to undertake self-management including focusing on illness needs (e.g tasks and skills required for the physical management of the condition), activating resources to assist with managing the condition and living with a chronic condition (e.g coping and integrating the condition into life) [11]. Key facilitators and barriers identified in the framework that can affect the ability to self-manage and outcomes include ‘personal factors’, ‘health status’, ‘resources’, ‘environment’ and the ‘healthcare system’ [11]. The framework includes ‘proximal outcomes’ (including ‘behaviours’, ‘cognitions’, ‘symptom management’ and ‘changes in biomarkers’) and ‘distal outcomes’ (including improved ‘health status’, ‘individual outcomes’ [e.g. quality of life], ‘family outcomes’ [e.g. functioning] and ‘health care outcomes’ [e.g. utilisation]) that result from self- and family management [11].

In relation to endometriosis, previous reviews have focused on women’s experiences [12] or the impact of the condition [13]. These have reported some limited findings related to women trialling complementary or self-care activities to manage the disease [12, 13]. A recent narrative review reported self-management and ‘psychological-sexological’ interventions in patients with endometriosis [14]. The review concluded that the efficacy of the complementary therapies that were investigated requires further investigation in RCTs and highlighted the importance of a multi-disciplinary team in managing endometriosis. This study did not use standard systematic review methods [15], the search strategy only included the Medline database and references in ‘relevant articles’, and the search terms were restricted to specific self-care activities and neglected other aspects of self-management, including active decision making, patient-provider partnership, health literacy, and behaviour change.

To date, there has been no systematic review of all the components of self-management in relation to endometriosis. The aim was to describe the evidence about the impact of self-management on condition-specific health among women with endometriosis. The specific objectives were to determine:

- The aspects of self-management that women with endometriosis undertake to assist them to manage the condition.
- The association between self-management and health and wellbeing outcomes
- The barriers to and facilitators of self-management among women with endometriosis.

## Methods

### Search strategy

The review was designed to meet the PRISMA guidelines. A search strategy was developed based on detailed knowledge of the field and in consultation with an expert librarian. The Medline (using the Ovid platform), PsycInfo (using the Ovid platform), CinahlPlus, Web of Science core collection (WOS) and Scopus electronic databases were searched. Search terms were entered both as keywords and mapped to individual database subject headings (where appropriate). Endometriosis was searched using subject headings and keyword term endometrio*. A broad perspective on self-management was adopted to capture the multi-faceted nature of this concept and to collate evidence that might not have been labelled ‘self-management’. Self-management covers knowledge, behaviours, and activities so over 40 terms were included to capture all elements (see Additional file 1: Appendix A). Reference lists of included papers were manually searched to identify further suitable papers. The systematic review was registered with Prospero (CRD42016042028).

### Eligibility criteria

Inclusion criteria were: reports of investigations of any element of self-management; among women (at least 18 years) diagnosed with endometriosis and published in English in a peer-reviewed journal. All study designs using quantitative or qualitative methods were eligible for inclusion. Exclusion criteria were: investigations of women with pelvic pain or suspected (but not confirmed) endometriosis or data from third parties (e.g health professionals or partners).

### Study selection

A two-stage process for assessing eligibility for inclusion was undertaken. First, an initial search of the literature was undertaken to review the titles and abstracts to identify articles that potentially met the inclusion criteria. Second, the full-text articles were reviewed, and any uncertainties were discussed and agreed upon by consensus among the authors.

### Data extraction and quality assessment

Data elements extracted included: author, year, country of study, research aim, method, recruitment, sample size, sample characteristics, results relating to self-management, barriers or facilitators to self-management and relationship to health and wellbeing outcomes. The quality of papers was assessed using the Standard Quality Assessment Criteria for Evaluating Primary Research Papers (QualSyst) [16]. Both the qualitative and quantitative checklists were used and the highest attainable score for each is 1.0 (Additional file 2: Table S1 & Additional file 3: Table S2). The quality assessment was completed independently by two authors and consensus was achieved through consultation among them.

## Results

### Search results

**Fig. 1:**
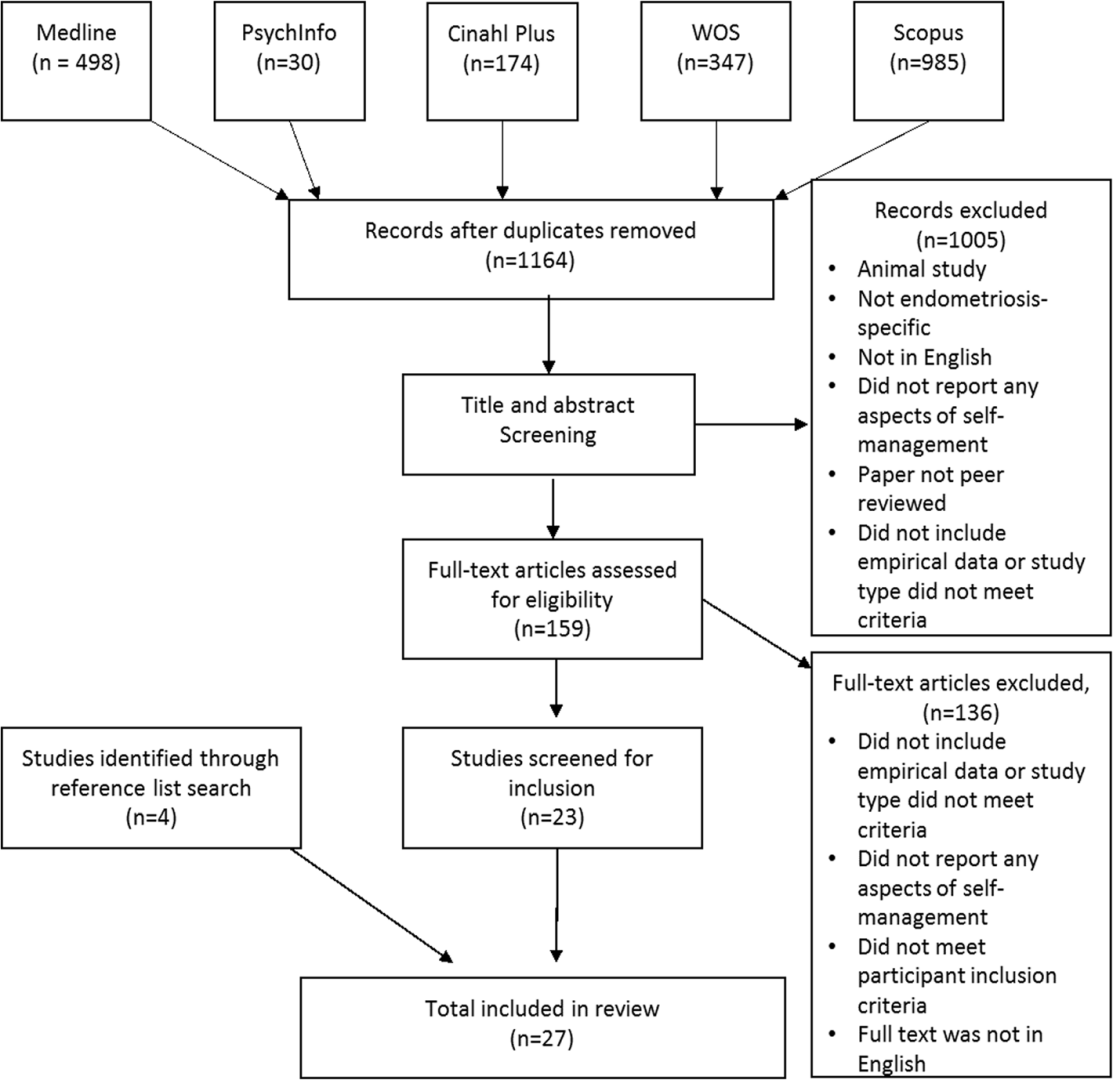
Systematic review flowchart

The search was conducted on the 13 April 2017 and yielded 2034 records, of which 159 full-text articles were retrieved and assessed for eligibility; 23 met inclusion criteria. A manual search of reference lists from these papers identified a further four records that were included in the review. This yielded a total of 27 papers reporting data from 19 studies (Fig. 1).

### Study characteristics

Of the included papers, two reported findings from RCTs of complementary therapies, seven reported surveys and 18 reported qualitative studies including focus groups or interviews. Most were conducted in high-income countries (US, UK, Sweden, Germany, Netherlands and Australia), but four papers reported findings from Brazil (two RCTs and 1 qualitative study) and South Africa (1 qualitative study), both upper-middle income countries.

Sample sizes for the RCTs were 22 [17] and 40 women [18]. The surveys had samples ranging from 23 to 4000 participants [19–24], one paper did not provide participant summary data [25]. Qualitative studies involved between 13 and 61 women [26–43], and one study also included the partners of women with endometriosis [26].

Reporting of demographic information varied (e.g. average age, age range, descriptive information). Most participants were aged in their 30’s. Participants were recruited through gynaecology, hospital, outpatient or GP clinics, or endometriosis support groups or a national endometriosis association. Of the papers that provided sufficient detail, the sample was predominantly educated and partnered. A summary of the study characteristics and the associated quality score is presented in Table 2.

**Table 2:** Summary of included studies

Author(s), Year Country	Aim	Study design, method, data source(s)	Sample Description Response Rate or Completion Rate (where applicable)	Self-Management Elements	Quality score
Randomised Controlled Trials					
Mira et al. 2015 Brazil [17]	Evaluate the effectiveness of Transcutaneous Electrical Nerve Stimulation (TENS) as a complementary treatment of chronic pelvic pain and/or deep dyspareunia among women with deep endometriosis and evaluate the effect on quality of life.	*Design:* Non-blinded RCT *Method:* Individuals randomised into two intervention groups (acupuncture-like TENS and self-applied TENS). No control group. Participants recruited through Women’s Hospital of the University of Campinas. *Data source:* VAS pain, EHP 30 (core and additional components) and study specific questions (sexual intercourse pain)	*Sample:* 22 women with deep endometriosis, experience pelvic pain &/or deep dyspareunia despite prescribed hormonal treatment (11 in each treatment group) *Characteristics:* Mean age 36.0 ± 7.1 Average age of diagnosis: 29.1 ± 7.8 Educated, employed, living with a partner *Completion rate:* 100% (no loss to follow up)	Self-care tasks	0.85
Gonçalves et al. 2017 Brazil [18] ■	The aim of this study was to compare chronic pelvic pain, menstrual patterns, and quality of life (QoL) between two groups of women with endometriosis: those who were assigned to an 8-week yoga intervention or to the usual standard of care.	*Design:* Mixed methods study including non-blinded RCT and interviews *Method:* Individuals randomised into two groups a yoga and control group (usual care). Participants recruited through endometriosis and physical therapy outpatient clinics of the University of Campinas Medical School. *Data source:* EHP30 (core and additional components), daily pain pattern (VAS) and study specific questions (menstrual pattern) Interview data reported in [34]	*Sample:* 40 women with endometriosis-associated chronic pelvic pain. 28 in the yoga group and 12 in control. *Characteristics:* Mean age 34.88 ± 6.7 Over 60% married/cohabitating 1/3 completed higher education 60% were employed *Completion rate:* 57% completed 8-week yoga program	Self-care tasks	0.81
Surveys					
Whitney 1998 United States [24]	To better understand the social support experiences of women with endometriosis	*Design:* Cross-sectional survey *Method*: Volunteers from an online endometriosis group were sent the survey. Open-ended questions – thematically analysed. Further details of the method not provided. *Data source:* Study specific questions (social support experience of women among spouse/partner, friends/extended family, others with endometriosis and health care providers)	*Sample:* 46 women with endometriosis from an online support group *Characteristics:* Sample described as predominantly from the ‘US, white, in their 30’s, well-educated and affluent’ (no further summary data provided). *Response rate:* 78% returned survey (46/59)	Patient-provider relationship, information seeking/knowledge, resource utilisation	0.44
Ballweg 2004 United States [19]	No aim stated. Appeared to be to compare registry data across two time periods.	*Design:* Cross-sectional survey – two time periods *Method:* Mailed out surveys to North American members of the US Endometriosis Society. Further details not provided. *Data source:* Details of questions not reported.	*Sample:* Members of US Endometriosis Society • Registry 1: 3020 participants (1980–1986) • Registry 2: 4000 participants (1998) *Characteristics:* Not reported. *Response rate*: Not reported.	Self-care tasks/behaviour change	0.30
Music 2005 United Kingdom [25]	No aim stated. Appeared to be to present the results of an evaluation of the UK Endometriosis Self-Management Course	*Design:* Not reported. Appears to be a cross-sectional survey evaluating a program. *Method:* Not reported. Appears to be 6 month follow up survey after a chronic disease self-management program conducted at Endometriosis UK. *Data source:* Details of questions not reported.	*Sample:* Not reported. Appears to be attendees of the program conducted by UK Endometriosis Association. *Characteristics:* Not reported. *Response rate:* Not reported.	Active participation, Self-care tasks Behaviour change Self-efficacy	0.17
Bodén et al. 2013 Sweden [20]	To investigate what type and level of support women diagnosed with endometriosis received from the school medical network and the nurses during their secondary (13–15 years old) and upper secondary school years (16–19 years old) and how it affected their quality of life.	*Design:* Cross-sectional online survey *Method:* Recruited participants through the Swedish Endometriosis Association. The association sent a letter with instructions to complete the survey online. The survey consisted of closed and open-ended questions. *Data source:* Study specific questions (background, history, and questions designed to get an account of participant experience). Specific details of the questions not provided in the paper.	*Sample:* 23 women finished schooling in last 10 years, who experienced symptoms during school years, later diagnosed with endometriosis (eligible if aged 18–26 years) *Characteristics:* Born between 1983 and 1990 Mean age of diagnosis 21 years (range 16–26 years) *Response rate:* 100% of those that were eligible.	Patient-provider relationship Information seeking/knowledge	0.56
Kundu et al. 2015 Germany [21]	To identify supporting and inhibiting factors on disease management to develop new support ideas.	*Design:* Cross-sectional survey *Method:* Participants recruited through newspaper, internet adverts and gynaecology clinics. Survey sent to participants prior to a training program for endometriosis. Reports results from open-ended questions that supplemented the training evaluation form. *Data source:* Study specific, open-ended questions about ‘coping’, what is lacking with managing the disorder and what could be improved.	*Sample:* 135 women with endometriosis, fluent in German *Characteristics:* Mean age 38.4 years (SD ± 8.0 years) (predominantly 31–45 age group) 68.9% married or cohabiting 61.5% university or technical college entrance *Response rate:* not reported.	Patient-provider relationship	0.75
Roos-Eysbouts et al. 2015 Netherlands [22]	To give an insight into characteristics of members of the Dutch Endometriosis Society members and evaluate their needs and expectations from the endometriosis society.	*Design:* Cross-sectional online survey *Method:* Survey emailed to all Dutch Endometriosis Society members that had an email address listed with the society. Survey featured multiple choice questions. *Data source:* Survey with study specific questions. 63 multiple choice questions relating to demographic characteristics, diagnosis, treatment, the impact of endometriosis and evaluation of patients’ needs and expectations from the endometriosis society.	*Sample:* 571 Dutch Endometriosis Society members *Characteristics:* The majority were in a ‘relationship, under 45 years of age, employed, and had completed tertiary education’. Response rate: 51% (571/1111)	Self-care/Behaviour change Information seeking/knowledge	0.78
Shoebotham &Coulson 2016 United Kingdom [23]	To examine the presence of therapeutic affordances as perceived by women who use endometriosis online support groups	*Design:* Cross-sectional online survey *Method:* Link to survey posted on three online endometriosis support groups (after group moderator approval). *Data source:* Study specific questions including demographic characteristics, use of online support groups, support group motives, experiences and coping. Paper reports results from a thematic analysis of open-ended questions.	*Sample:* 69 women in an online support group *Characteristics:* Mean age of 34.2 (range 19–50 years) Majority residents of UK (65.2%) or the US (21.7%) *Response rate:* not reported, no indication of total members of the groups.	Information seeking/knowledge	0.69
Qualitative studies – involving focus groups and interviews					
Cox et al. 2003 Australia [28]◆	Study: To identify women’s needs for information related to laparoscopy for endometriosis. Paper: the experience of health care and the use of complementary therapies to manage symptoms.	*Design:* Mixed methods (cross-sectional survey and focus groups) – paper reports findings from focus group *Method:* Women were recruited from Victorian Endometriosis Association and Epworth Hospital database. Focus group participants were recruited from respondents that completed the survey (survey *n* = 670). Three groups were conducted face-to-face and two were conducted over the telephone. *Data source:* Subject areas or interview guide not provided. Results relate to the struggle of living with the disease, becoming assertive and use of complementary/alternative therapies.	*Sample:* 61 women with endometriosis *Characteristics:* predominantly Victorian city sample Age range 20–64 (provided in categories) Largest age group 30–34 years	Active participation Informed decisions Monitoring symptoms Patient provider relationship	0.45
Cox et al. 2003 Australia [27]◆	To identify the information needs of women facing laparoscopy for endometriosis	*Design and method:* Refer to [28] *Data source:* Women invited to discuss: What information they would like to receive or contribute about endometriosis relating to: • Nature of disease • Experience of living with endometriosis • Experience with diagnosis and treatment	*Sample and characteristics:* Refer to [28]	Active participation Informed decisions Monitoring symptoms Patient provider relationship	0.75
Jones et al. 2004 United Kingdom [35]	To identify and understand, from the patient’s perspective, the areas of HRQoL that are affected by endometriosis and to address the benefits of using a qualitative methodology for item generation in the development of disease-specific health status questionnaires.	*Design: S*emi-structured interviews *Method:* Recruited through an outpatient clinic at the Women’s Centre, John Radcliffe Hospital, Oxford. Interviews conducted in a research facility at the hospital. *Data source:* Women were asked: ‘Please feel free to say anything about what your life has been like with the condition’. Prompts relating to QOL were derived from the literature.	*Sample:* 24 women diagnosed with endometriosis *Characteristics:* Mean age 32.5 (Range 21.5–44 years) 12 married, 3 separated, 2 cohabiting, 4 long-term relationship (not living together), 3 single. 14 were nulliparous (6 undergoing IVF)	Self-care/Behaviour change Monitoring symptoms	0.95
Denny 2004 United Kingdom [29] ❖	To explore the lives of women with endometriosis	*Design:* In-depth interviews *Method:* Recruited through self-help groups, gynaecology department at a local hospital and snowballing. 20 interviews conducted primarily in women’s homes *Data Source:* Story-telling approach – e.g. first experience of symptoms associated with endometriosis. Follow up questions on pain, social relationships, working life, sickness relationship with health professionals.	*Sample:* 20 women diagnosed with endometriosis *Characteristics:* Mean age 33 years (Range 20–47 years) Majority ‘middle class, white British’	Information seeking Knowledge Patient-provider relationship	0.75
Denny 2004 United Kingdom [30] ❖	To explore women’s experience of living with endometriosis.	*Design, Method, Data Sources*: Refer to [29]. At the time of publication 15 interviews had been conducted.	*Sample:* 15 women diagnosed with endometriosis *Characteristics:* Demographic information not provided in this paper	Self-care activities	0.80
Strzempko Butt & Chesla 2007 United States [26]	To investigate responses in the couple’s relationship to living with chronic pelvic pain (CPP) from endometriosis. Takes into account the socio-cultural context.	*Design:* In-depth interviews *Method:* Recruited through clinics (public and private) and endometriosis groups. Included individual interviews with women, partners and couple interviews. Data source: Interviews started with the question ‘Please tell me about something that has happened recently in relation to your endometriosis that was difficult for you or your partner. Please just tell me the story as it happened’. Additional prompts around illness understanding, symptom experience, relational responses to CPP.	*Sample:* 13 women with endometriosis that experience chronic pelvic pain for at least 6 months and 13 male partners *Characteristics:* lived together mean 6 years (range 1 to 23) mainly childless (two couples had 1 child) Mean age women 34 (range 23 to 48 years) male partners mean age 38 (range 24 to 50 years) mainly employed (92% women, 84% men) health insurance (all women, 85% of partners) 60% were European American almost 50% had a household income of US $100,000 or above	Self-care activities	0.85
Denny & Mann 2007 United Kingdom [32] ●	To understand the impact of dyspareunia on women’s lives	*Design:* Semi-structured interviews *Method:* recruited from an endometriosis outpatient clinic. Interviews conducted in an acceptable place for women. Adopted a storytelling approach. *Data source:* Invited to ‘tell their story of living with endometriosis from the time they first experienced symptoms’. 14 spontaneously discussed painful intercourse – follow up question for those that didn’t.	*Sample:* 30 women diagnosed with endometriosis *Characteristics:* Mean age 31 (range 19–44 years) Majority ‘White British’, from ‘social classes 1–3’ married or cohabiting (20/30) all heterosexual women with children (11 + 2 pregnant)	Self-care activities	0.80
Manderson et al. 2008 Australia [36] ○	To explore whether and how women’s experience of gynaecological or reproductive health problems or conditions impacted their gendered, social and personal identities.	*Design:* In-depth interview *Method:* Substudy within a larger study (paper presents information from women with endometriosis). Recruited through community newspapers, noticeboards, and snowball sampling. Interviews conducted in place of women’s choice (mostly own home) *Data Source:* Details of questions not provided. Results related to pathways of treatment-seeking among women with endometriosis.	*Sample:* 40 women with endometriosis, living in Victoria *Characteristics:* Mean age 45.5 (range 20 to 78 years) 88% Australian born Range of social and economic backgrounds, lived in geographically diverse areas 30% not in paid employment, 50% of women were in managerial or professional occupations	Self-care activtities	0.75
Markovic et al. 2008 Australia [37]○	To enrich understanding of the relationship between the patient’s socio-demographic background and health-related phenomena	*Design, method:* Refer to [36] At the time of publication 30 interviews had been conducted. *Data source:* Details of questions not provided. Results relate to illness narratives of endurance and contest.	*Sample:* 30 women with endometriosis, living in Victoria *Characteristics:* 25 Australian born Mean age 43.9 years (range 20–78) Most resided outside of metropolitan areas 2/3 married or defacto relationships	Active participation, self-care/Behaviour change Informed decisions Monitoring symptoms Patient provider -relationship Information seeking/knowledge	0.75
Denny & Mann 2008 United Kingdom [33] ●	To explore the experience of women with endometriosis in the primary care setting.	*Design:* Semi-structured interviews *Method:* Recruited through an endometriosis clinic at a women’s hospital. Interviews conducted in own home or clinic. *Data source:* Women were invited ‘to tell their story from the first experience of symptoms’. 17 spontaneously mentioned relationships with GPs – additional prompts for those that didn’t.	*Sample:* 30 women with endometriosis *Characteristics:* mean age 31 (range 19 to 44) 27 classed as ‘socio-economic class 1–3’ 27 were ‘white British’	Patient – provider relationship	0.75
Seear 2009 Australia [40] ⟡	To explore the experiences of Australian women living with endometriosis – focus on becoming expert patients.	*Design: S*emi-structured interviews *Method:* Recruited through snowball sampling and newsletter of support group. ‘Expert patients’ was not a focus of study but emerged as a key theme. *Data source:* Questions explored: diagnosis, treatment, the doctor-patient relationship, self-help, causation and illness experiences.	*Sample:* 20 women with endometriosis *Characteristics:* Mean age 34 years (range 24–55) Majority married or in a relationship, mainly Anglo-Celtic and tertiary educated 10/20 members current or previous members of a support group	Self-care tasks Patient – provider relationship Information seeking/knowledge	0.60
Seear 2009 Australia [41] ⟡	To explore the experiences of 20 Australian women living with endometriosis – focus on menstrual etiquette/stigmatisation	*Design, method, data source:* Refer to [40]	*Sample and characteristics:* Refer to [40] Mean age of diagnosis 27 years	Self-care/Behaviour change Patient-provider relationship	0.55
Seear 2009 Australia [42] ⟡	To examine non-compliance with health advice among women with endometriosis.	*Design, method, data source:* Refer to [40] Non-compliance was not a focus of the study but raised spontaneously by some of the participants	*Sample and characteristics:* Refer to [40]	Active participation Self-care/Behaviour change Informed decisions Patient-provider relationship	0.55
Denny 2009 United Kingdom [31]●	To explore women’s experience of living with endometriosis in a prospective study over a 1 year period.	*Design:* Repeated semi-structured interviews *Method:* Recruited from an endometriosis clinic at a women’s hospital. Interviews conducted at a place chosen by the participant. Interviews conducted upon recruitment and 1 year after. Volunteer sample kept a diary of experiences of endometriosis over one menstrual cycle. *Data source:* Interview started with ‘tell me about living with endometriosis from the time you first experienced pain’. Follow up questions on the impact on life, relationship.	*Sample:* 30 women recruited – 27 were interviewed at 1 year 19 women were asked to keep a diary – only seven completed and returned it *Characteristics:* 20 women were married or cohabitating 27 described ethnicity as ‘white British’, *Response rate:* 90% (27/30 completed interview at 1 year)	Self-care/Behaviour change Patient-provider relationship	0.90
Moradi et al. 2014 Australia [38]	To explore women’s experiences of the impact of endometriosis and whether there are differences across three age groups.	*Design: S*emi-structured focus groups *Method:* Recruited from an endometriosis clinic at a Canberra public teaching hospital and GP in community and information evening. Conducted 10 focus groups with 3 to 4 participants per group. Groups were split into age groups: Group 1 (16–24 years), Group 2 (25–34 years) and Group 3 (35 years and above). *Data source:* interview guide developed with two main questions ‘How are women’s experiences of living with endometriosis?’ & ‘How does endometriosis affect women’s lives?’	*Sample:* 35 women with endometriosis *Characteristics:* mean age was 31.1 ± 10.4 years (range 17–53). Most (30 out of 35) were Australian born, Most were married or had partners	Self-care/Behaviour change Patient-provider relationship	0.85
Gonçalves 2016 Brazil [34] ■	This study sought to understand the meanings that women with pain-associated endometriosis attribute to an 8-week yoga program regarding their bodily experiences with the practice and their perceived potential benefits	*Design:* Mixed methods (RCT and semi-structured interviews) *Method:* Focus of paper is on interviews. Purposive sampling – volunteers who completed the 8-week yoga program from RCT. *Data source: I*nterview guide – endometriosis symptoms and yoga practice	*Sample:* 15 women who had completed the 8-week yoga program *Characteristics:* aged 24–49 years, more than half were married, more than half had completed high school or higher education. More than half were working during the study.	Self-care	0.70
Roomaney & Kagee 2016 South Africa [39]	To explore how patients in a resource-constrained setting coped with living with endometriosis.	*Design:* Semi-structured interviews *Method:* Participants recruited from an obstetrics & gynaecology department at a Cape Town hospital. Interviews conducted at the researcher’s office, participants’ homes or location selected by participants. Interviews conducted English or Afrikaans (participant preference). *Data source:* Interview guide started with ‘can you tell me about your experience with endometriosis’ – 13 prompts to explore this experience.	*Sample:* 16 women diagnosed with endometriosis *Characteristics:* Mean age 33 years (range 23–42 years). Eight were married, 3 divorced, 5 single Majority employed 9 were employed full-time, 3 were employed part-time, 3 were unemployed and 1 was a student.	Self-care/Behaviour change Information seeking/Knowledge	0.85
Young et al. 2016 Australia [43]	To increase understanding of women’s experiences of endometriosis from their perspective.	*Design:* In-depth interviews *Method:* Recruited through women’s health magazine and event, queer-friendly organisations & cultural services. Interviews conducted in person (home/research facility) or via telephone *Data source:* Interview guide started with ‘Please tell me about your experience of endometriosis. You can start from whatever point you like and include whatever you find necessary’ (additional prompts explored experience)	*Sample:* 26 women diagnosed with endometriosis in Victoria *Characteristics:* Majority aged in 30’s, born in Australia, completed an undergraduate degree live with partner and identified as heterosexual.	Patient-provider relationship	0.90

■♦◆❖●○⟡ Symbols indicate data generated by the same study. *TENS* Transcutaneous electrical nerve stimulation, *VAS* Visual Analogue Scale, *EHP30* Endometriosis health profile, *RCT* randomised controlled trial, *CPP* chronic pelvic pain, *f2f* face to face, self-management elements (Table 1)

### Quality assessment

Study quality was assessed using QualSyst [16] with the highest possible total score of 1 (Additional file 2: Table S1 & Additional file 3: Table S2). In addition, evidence of human research ethics committee approval was assessed. Of the 27 papers included, only 19 reported approval from a formally constituted institutional human research ethics committee and seven did not; one paper reported findings from a study that indicated in an associated paper that ethics approval had been obtained.

#### Quality of RCTs

Two RCTs investigated complementary approaches to endometriosis pain-management including the use of acupuncture-like and self-applied Transcutaneous Electrical Nerve Stimulation (TENS) [17] and an 8-week yoga program [18]. The quality scores were 0.81 and 0.85. Both used the EHP30 a validated endometriosis specific quality of life measure, and study-specific questions to measure outcomes [17, 18]. There was too few data for a meta-analysis.

Mira et al. [17] compared two different types of TENS, but it lacked a control group (e.g. sham TENS). The use of the acupuncture-like TENS machine required interaction with a physiotherapist which may have influenced some domains of the EHP30, among women in this group and is a limitation of the study [17]. All participants were retained and assessed at trial endline.

Goncalves et al. [18] compared participants of an 8-week yoga program to a control condition of no yoga practice. Sample size was calculated from prior research, but there was a high loss to follow up and only 57% of the yoga group completed the full 8-week program [18].

#### Quality of survey research

All the surveys were cross-sectional and quality scores ranged from 0.17 to 0.78 [19–25]. Papers reported descriptive statistics [19, 20, 22, 25] or qualitative analysis (e.g thematic or content analysis) of open-ended survey responses [20, 21, 23, 24]. No studies reported multivariable analyses and one did not report the sample size [25]. Two studies [19, 25] did not provide sufficient detail about methods, data collection tools or participants.

#### Quality of qualitative studies

The qualitative studies used interviews or focus groups to collect data with quality scores ranging from 0.45 to 0.90. Only three [26, 31, 35] considered reflexivity, a core component of qualitative research that acknowledges the role that researchers play as a part of the world they study and its influence on data that are collected [44]. Four studies neglected to report verification procedures (e.g. triangulation, peer review, member checks) associated with analysis that are used to contribute to the rigour of qualitative studies [27, 40–42]. Information about the context of the study, recruitment strategies and question guides was limited, reflecting lower scores on these criteria (Additional file 3: Table S2).

### Self-management findings

A total of nine papers expressly used the term ‘self-management’ (three papers reporting findings from surveys [21, 23, 25] and six qualitative papers [36–40, 42]). Formal definitions were not provided with the exception of Roomaney and Kagee [39] who defined self-management as ‘steps taken by the participants to alleviate the symptoms of endometriosis’. However, this definition is more consistent with the definition of ‘self-care’ than that of self-management.

### Aspects of self-management

No study examined all 11 elements of self-management, rather they explored experiences with endometriosis and the needs of women with endometriosis. Findings related to self-management were incidental and were associated with one or more of the elements of self-management (see Table 1).

#### Relationship with health providers

Few studies investigated whether a ‘partnership’ between the woman with endometriosis and the health care provider had been established, rather women’s experiences with healthcare providers (e.g. doctors or nurses) were explored [20, 21, 24, 27–31, 33, 35, 37, 38, 41–43].

Positive experiences were associated with the technical competence of the practitioner in endometriosis [21, 24], a ‘person-centred’ approach [24, 43], pursuit of investigation or treatment options [37, 41], referral to a specialist [33] and effective communication (sharing information and knowledge) [21, 43]. Some women’s medical professionals prompted them to take an active role in their healthcare [40], and other women reported that their GP was an ‘ally’ in the treatment of their disease [29]. Positive experiences with health care providers were associated with a feeling of greater ‘control’ [24] and a sense of relief as the provider could explain the disease and available options [27].

Four papers reported that women changed providers if they were not satisfied with the care they had received [27, 28, 37, 43]. This sometimes required multiple presentations to different health providers [37]. Negative experiences were reported more commonly than positive experiences and were associated with providers lacking knowledge of the condition [20, 27, 29, 33, 35, 42], normalising symptoms as part of menstruation [29–31, 33, 38], having poor communication skills [20, 27]; not providing information or providing information not appropriate to women’s needs [27, 43], reluctance to refer to specialists [27, 33] and difficulty accessing qualified specialists [38]. Some healthcare providers were described as demonstrating negative attitudes such as doubting women’s reports of symptoms, not taking the individual seriously, being dismissive or unsympathetic, or criticising women for not exerting enough effort to manage their endometriosis [20, 27, 29, 31, 33, 37, 38, 42]. When the relationship with the health provider was poor women reported they felt neglected [20] or felt they were being dismissed which affected their self-esteem [27].

#### Information seeking

Eight papers reported findings in relation to women seeking information about endometriosis, treatments, research, specialists, natural therapies, other management options and surgery [20, 22–24, 29, 37, 39, 40]. Information sources included wide reading, the internet, and support groups or endometriosis associations [20, 22–24, 37]. Being informed about the condition enabled women to ‘take charge’ and empowered them to make decisions about their health [28]. However, information-seeking also was related to feeling overwhelmed, overloaded, contributed to anxiety about the condition and some women experienced difficulty with conflicting information [40].

Four papers reported findings from women who joined support groups or endometriosis-specific associations [22–24, 40]. The main reasons for joining these groups was to obtain support, exchange self-help advice (e.g diet and supplements) and the names of appropriate physicians [22–24]. However, Shoebotham & Coulson [23] concluded that some women had concerns over the quality of information that was shared in these groups. Support groups provided an opportunity to connect with others with the disease, which alleviated feelings of isolation [23, 24, 39]. Positive experiences of groups included improved knowledge, empowerment and quality of life [22, 23]. Providing support to others was beneficial and rewarding [23, 24].

#### Monitoring symptoms

Four papers described how some participants recorded and monitored symptoms of their endometriosis with the intention to provide evidence for medical professionals [28, 37], locate patterns (e.g cyclical nature of symptoms) [27], or to predict when symptoms would be severe [35]. Cox et al. [28] reported that recording symptoms provided women with validation that their symptoms where physiological and linked to their menstrual cycle.

#### Decision making

Four papers reported findings related to ‘taking control’ or taking an active role in treatment and management decisions [27, 28, 37, 42]. Cox et al. [27, 28] reported that women were informed about their condition and exercised control over decisions around providers. Two papers reported that women were assertive with health providers, for example by providing proof of the reality of their condition through recorded symptoms [28, 37]. Four papers highlighted that treatment decisions were taken seriously by women, for example about whether to proceed with surgery, or whether to start or continue with medical treatments [27, 28, 37, 42]. Cox et al. [27, 28] discussed that women engaged in goal setting such as being drug-free or achieving good pain management or getting ‘off the medical roundabout’. Two papers described women becoming ‘experts in their own care’ [31, 40]. ‘Taking control’ of their health and decisions around health enabled women to feel empowered, liberated and allowed them to ‘tune in’ and listen to their bodies [27, 28, 40].

#### Self-care tasks/behaviour change/complementary therapies

Self-care tasks, complementary therapies and behaviour change were the most widely reported aspects of self-management. Eighteen studies reported accounts of different self-care activities [17–19, 22, 25, 26, 28, 30–32, 34–42]. Cox et al. [28] reported that three women of the 61 included in the study had replaced medical management with self-care and alternative therapies. Table 3 outlines the lifestyle changes, cognitive approaches, behaviour change and pain management approaches that women with endometriosis employed.

**Table 3:** Summary of self-care and behaviour change activities

Activity	Description
Lifestyle changes	• changing diet [19, 26, 28, 31, 38–40]
	• taking herbs and vitamins [28, 40, 42];
	• exercise [19, 28, 38, 40] (for example yoga [18, 34, 40], Pilates [19, 42], stretching [39], chi-gong [19])
	• avoiding chemicals (for example making own cleaning products [40], or eating organic food [42])
	• quitting smoking [38].
Cognitive approaches	• ‘positive thinking’ [28, 39],
	• meditation [26, 40],
	• accepting the disease and learning to live with the condition [39],
	• using self-talk to overcome pain [39],
	• evoking spirituality (praying or others’ prayers) [39]
Behaviour change	• limiting activity: resting [26, 35, 37, 39]; not attending social functions [30, 35, 38, 39]; staying at home or close to home [26, 38, 39], reducing exercise /sport [26, 38, 39]; getting good sleep [38]
	• changing work conditions: taking leave [30, 35] or resigning from work [30], working from home [26], reducing work hours [42]
	• sexual activity: avoiding intercourse [32, 34, 35, 38, 41], adapting sexual position [26, 30, 32] or exploring alternatives to penetrative sex [32], masturbating to orgasm to alleviate pain [36]
	• scheduling activities for when pain is not as severe [31, 35, 39]
	• reducing stress [40]
	• enlisting assistance from others [26]
	• managing heavy bleeding: wearing multiple feminine hygiene products [41]
Pain management	• taking analgesics [22, 31, 35, 36, 39, 41],
	• carrying pain medications [26],
	• using heat [31, 36, 37, 39],
	• massage [34, 36],
	• TENS machine [17, 31, 36];
	• breathing techniques or relaxation exercises [34, 36, 37, 39]

Lifestyle changes can benefit health [38] but could also result in too much emphasis, lack of enjoyment and changes becoming physically and emotionally demanding [40, 42]. Seear [40] reported that few women experienced any health benefits following the implementation of these self-care practices. Women reported feeling less confident, or being constantly worried about the condition or resulted in less social contact with other people [30, 35, 38], as a result of staying at home or cancelling social events. Avoiding intercourse due to pain resulted in women reporting guilt or inadequacy [35], negative impacts on their relationship [32, 38] and low self-esteem [32].

Seven of the papers reported that women with endometriosis pursued complementary therapies including candidiasis treatment, counselling, acupuncture, homeopathic treatment, naturopathy, lymphatic drainage, massage, Chinese herbal medicine, Reiki, healing touch, aromatherapy, spiritual healing, TENS and Yoga. [17–19, 22, 26–28].

### Health and wellbeing outcomes

Four studies assessed health and wellbeing outcomes (two RCTs investigating the use of TENS and yoga respectively) and two surveys. The other studies did not actively measure health and wellbeing outcomes however, some incidental findings were discussed during the course of investigating women’s experiences with endometriosis and the needs of women with endometriosis, which have been summarised in the previous section.

#### Transcutaneous electrical nerve stimulation

Both types of TENS machines (acupuncture like and self-applied) provided symptomatic pain relief for chronic pelvic pain and deep dyspareunia [17]. Pain with defecation improved with the use of the acupuncture-like TENS [17]. The results from the intervention showed statistically significant improvements in the EHP30 domains for pain, control and powerlessness, emotional wellbeing, social support, self-image, work, sexual intercourse and treatment [17].

#### Yoga

There was a statistically significant positive association between yoga and pain relief and quality of life across EHP30 domains of pain, control and powerlessness, emotional wellbeing, self-image, work and treatment. There was no difference between the two groups regarding menstrual patterns. Health and wellbeing outcomes were further elaborated on in the qualitative component of this study [34]. Participants reported that the yoga program was beneficial for controlling pelvic pain through relaxation and breathing techniques [34]. Participants were able to implement breathing and relaxation techniques on their own to respond to pain [34]. Women who completed the yoga program reported an increased level of body awareness and the program also offered social support by connecting with other women with the disease [34].

#### Self-management program

One study reported survey findings from an evaluation of a self-management program [25]. The program is a peer-led program involving six weekly sessions, each lasting two and a half hours, covering cognitive pain therapy, dealing with feelings of anger, dealing with fear and frustration, communicating effectively with health care professionals, goal setting, and action planning. After the program participants reported greater confidence, improved quality of life, less anxiety, felt better prepared, greater control in decisions, had reduced the use of pain medications and improved relationships with their health care provider [25].

#### Self-care/complementary therapies

Only one survey investigated the efficacy of self-care activities or complementary therapies [19]. The study asked whether a series of specific alternative approaches were ‘helpful’ but did not elaborate on what was meant by the term ‘helpful’ [19]. In Ballweg’s study [19] 63% of participants reported that exercise was helpful, 62% indicated a change in diet, 65% reported Candidiasis treatment; 59% counselling; 56% acupuncture and 56% indicated vitamin and mineral supplements were ‘helpful’.

### Facilitators and barriers to self-management

None of the studies specifically investigated facilitators and barriers to self-management. However, some incidental findings were reported in 10 papers [21, 27, 28, 32, 35, 37–40, 42].

#### Facilitators

Two facilitators were discussed in the papers which contributed to active engagement in decision making or instigating behaviour change. Four papers reported that experiences with health professionals prompted women to ‘take control’ of their health, some due to support of providers, whilst others had negative experiences with medical intervention or felt that they had no choice and needed to take action [27, 28, 31, 40]. Women’s partners were supportive of behaviour change to avoid pain, for example by avoiding sexual intercourse [32].

#### Barriers

Roomaney and Kagee [39] reported that women who lacked knowledge about the disease upon diagnosis needed to learn more about it in order to manage it. A ‘lack of control’ and sense of powerlessness was highlighted by Jones et al. [35] as some women reported that they were not able to control the symptoms of endometriosis, which may affect their ability to implement activities to manage the disease. At times women reported that endometriosis symptoms hinder attempts to undertake activities that may be beneficial to their health [38, 42] or that it was not always practical to make changes to their lifestyle (e.g. exercise) [42] and that the costs of products or services were prohibitive (e.g. alternative treatment, medicines, diet) [28, 35, 40, 42]. Place of residence can be a hindrance to making changes to diet [42] (e.g growing own food) or accessing health professionals [37]. Deficiencies in health professionals’ knowledge, empathy, and communication skills were key barriers to the management of endometriosis [21].

## Discussion

Evidence from this review indicates that self-management among women with endometriosis is an emerging field of research. Overall, this body of literature was assessed to be of moderate quality. However, many of the participants in these studies were recruited through a clinic, endometriosis support group or national endometriosis association, which may have resulted in recruitment of participants that were more engaged, or with complex cases or who experience more symptoms compared with women with endometriosis in the general community. Therefore the results should be interpreted with caution as they may have limited generalisability to the wider population of women with endometriosis. Differences in study-specific data collection methods of included studies made comparisons of results difficult.

The evidence from this review highlights that some aspects of self-management among women with endometriosis such as self-care activities, use of complementary therapies and relationship with providers have been investigated, but the remaining elements of self-management (Table 1) require further investigation. Further, no paper examined all elements of self-management and there were no randomised controlled trials (RCTs) of comprehensive self-management programs compared to usual care among women with endometriosis.

There remains a need for further research into self-management in endometriosis that is informed by a theoretical framework (e.g. the self- and family management framework) [11] and complete definition of self-management. There was limited evidence on health and wellbeing outcomes and facilitators and barriers to self-management, which suggests that these aspects warrant further investigation.

### Strengths and limitations of the review

This review had a number of strengths. First, the search strategy was constructed with an expert librarian and was based on a thorough review of self-management definitions. Second, the systematic review was guided by a published protocol (registered with Prospero CRD42016042028). Third, the quality assessment was directed by a standardised quality assessment tool and two authors independently reviewed the quality of the papers [16]. A limitation of the review is that only studies reported in English were included, and there may be relevant studies in other languages that reported culture-specific information but were missed.

### Implications

#### Implications for women

Findings from the review suggest that both complementary therapies [17–19, 22, 26–28] and self-care activities (tasks an individual performs at home in order to manage the symptoms of a condition) [7] are widely used to manage the symptoms of endometriosis. However there was limited evidence as to the efficacy of complementary therapies and self-care activities for managing endometriosis. The findings from two RCTs provide preliminary evidence that Yoga and TENS may be useful for women with endometriosis as both these therapies were associated with significant improvements in physical and psychosocial domains of the EHP30 [17, 18]. Evidence for the utility of self-care practices was mixed Ballweg [19] reported that changing diet, for example, was ‘useful’ but in Seear’s study [40] few women reported experiencing any health benefits following the implementation of self-care practices. This suggests that women may need to be discerning in the types of therapies they choose and further studies are required to investigate the relationship between additional complementary therapies, self-care activities and improvements in health and wellbeing.

#### Implications for providers and health care

Effective patient-provider partnerships are crucial to successful self-management in chronic diseases [7, 9]. Health professionals’ knowledge, empathy, and good communication skills assist women to manage their condition but are not always adequate [21] and may prompt a change in provider [27, 28, 37, 43], sometimes multiple times [37]. Whilst no study expressly measured the health and wellbeing impacts of the patient-provider relationship, incidental findings suggest that positive relationships provide women with a ‘feeling of control’ over the condition [24] and may prompt women to take an active role in their healthcare [29, 40]. Conversely, poor relationships characterised by minimisation of symptoms, or dismissiveness can engender feelings of neglect and abandonment, and damage to self-esteem [20, 27].

There is a need to support providers to integrate practices which promote positive patient-provider partnerships. The findings suggest a need for endometriosis-specific education for providers caring for women with this condition and integration of patient-centred practices. These practices may include providing education to the patient about the condition, identifying problems from the patient’s perspective and creating a plan that includes goal-setting and strategies to overcome problems associated with managing the condition [45]. This may necessitate a longer consultation in order to facilitate these discussions, which concurs with Oldroyd et al. [46] findings from GP’s that reported longer consultations for chronic disease care are required.

Creating an individualised plan for managing the condition may be a useful mechanism to prompt discussion of specific activities or alternative approaches that women are using to manage the symptoms, given the variety of self-care activities and complementary therapies reported by women with endometriosis in the literature. Providers are encouraged to foster an open discussion, avoid being dismissive or critical for women using these therapies, but encourage women to seek evidence-based therapies.

In a review of chronic disease self-management interventions, Barlow and colleagues [6] found that compared to usual care, self-management interventions are effective in improving knowledge, self-efficacy, the performance of self-management tasks and some aspects of health status [6]. No RCT has been conducted to determine the efficacy of a chronic disease self-management intervention for women with endometriosis. However, Music’s evaluation of a self-management program [25] highlighted that after the program participants reported greater confidence, improved quality of life, less anxiety, felt better prepared, greater control in decisions, had reduced the use of pain medications and improved relationships with their health care provider. Whilst the findings should be interpreted with caution as this study was of low quality as there was insufficient detail provided about methods, data collection tools or participants, it would suggest that an RCT that compares a self-management intervention to usual care among women with endometriosis may be warranted.

## Conclusion

Self-management is an emerging area of research in endometriosis. The results of this review provide evidence that self-care activities, use of complementary therapies and positive patient-healthcare provider relationships are important components of managing endometriosis. However, further purposeful research is warranted using a clear definition of and comprehensive theoretical framework for self-management. More research is required on health and wellbeing outcomes and facilitators and barriers to self-management among women with endometriosis. An investigation on the efficacy of a self-management program for women with endometriosis is also warranted, given the benefits that have been observed for these programs in other chronic diseases.

## Additional files

Additional file 1: Appendix A. Search strategies for databases. (DOCX 29 kb)

Additional file 2: **Table S1.** Quality assessment scores for the papers involving quantitative studies. (DOCX 18 kb)

Additional file 3: **Table S2.** Quality assessment scores for the papers involving qualitative methods. (DOCX 20 kb)

## Data Availability

All data generated or analysed during this study are included in this published article [and its supplementary information files].

## Abbreviations

GP: General Practitioner
PRISMA guidelines: Preferred reporting items for systematic reviews and meta-analyses
RCTs: Randomised controlled trial
TENS: Transcutaneous Electrical Nerve Stimulation
