# Supplementary material for: Self-management in condition-specific health: a systematic review of the evidence among women diagnosed with endometriosis
Source: BMC Womens Health. 2019 Jun 19;19:80. doi: 10.1186/s12905-019-0774-6 (PMC6585070; doi:10.1186/s12905-019-0774-6)
Supplement: Supplementary file 1 — Appendix A. Search strategies for databases. (DOCX 29 kb) [file 12905_2019_774_MOESM1_ESM.docx]

**Appendix A – Search strategies for databases**

**Database: PsycINFO <1806 to March Week 3 2017>**

**Search Strategy:**

--------------------------------------------------------------------------------

1 endometrio*.mp. (217)

2 Self-Care Skills/ or Self-Management/ or self care.mp. or self manag*.mp. (18892)

3 Self-Help Techniques/ or self help.mp. (9111)

4 expert patient*.mp. (92)

5 Health Behavior/ (22727)

6 (health behavior* or health behaviour*).mp. (28875)

7 self efficacy.mp. or Self-Efficacy/ (35573)

8 self concept.mp. or Self-Concept/ (48635)

9 self confidence.mp. or Self-Confidence/ (7455)

10 self help group*.mp. (1936)

11 social support.mp. or Social Support/ (53418)

12 support group*.mp. or Support Groups/ (8557)

13 client participation/ (1712)

14 client participation/ or patient* participat*.mp. or client* participat*.mp. (4500)

15 consumer participat*.mp. (278)

16 problem solving.mp. or Problem Solving/ (47756)

17 decision making.mp. or Decision Making/ (98654)

18 Health Attitudes/ or attitud* to health.mp. (10018)

19 Client Attitudes/ (15128)

20 (health attitude* or patient* attitude* or client* attitude*).mp. (25419)

21 professional patient relation*.mp. (76)

22 physician patient relation*.mp. (913)

23 Interpersonal Relationships/ or interpersonal relation*.mp. (25905)

24 communication.mp. or COMMUNICATION/ (223120)

25 (patient centered or patient centred).mp. (4501)

26 (cooperative behavior or cooperative behaviour).mp. (1180)

27 cooperat*.mp. (47781)

28 Cooperation/ or Collaboration/ (20664)

29 collaborat*.mp. (69449)

30 partnership.mp. (12479)

31 health partner*.mp. (211)

32 consumer health information.mp. (99)

33 health knowledge.mp. or Health Knowledge/ (7437)

34 health information.mp. (5071)

35 health education.mp. or Health Education/ (16068)

36 health promot*.mp. or Health Promotion/ (26933)

37 patient* educat*.mp. (3238)

38 information seeking.mp. or Information Seeking/ (4792)

39 Health Literacy/ or health litera*.mp. (3941)

40 Client Education/ or client* educat*.mp. (3576)

41 self educat*.mp. (290)

42 Help Seeking Behavior/ or help seeking.mp. (8215)

43 treatment seeking.mp. (3711)

44 health care utili?ation.mp. or Health Care Utilization/ (14643)

45 Health Care Seeking Behavior/ or health care seeking.mp. (3693)

46 2 or 3 or 4 or 5 or 6 or 7 or 8 or 9 or 10 or 11 or 12 or 13 or 14 or 15 or 16 or 17 or 18 or 19 or 20 or 21 or 22 or 23 or 24 or 25 or 26 or 27 or 28 or 29 or 30 or 31 or 32 or 33 or 34 or 35 or 36 or 37 or 38 or 39 or 40 or 41 or 42 or 43 or 44 or 45 (719980)

47 1 and 46 (31)

48 limit 47 to english language (30)

***************************

**Database: Ovid MEDLINE(R) 1946 to Present with Daily Update**

**Search Strategy:**

--------------------------------------------------------------------------------

1 Endometriosis/ (19306)

2 endometrio*.mp. (27873)

3 Self Care/ or self manag*.mp. (34267)

4 self help.mp. (16221)

5 expert patient*.mp. (184)

6 Health Behavior/ (42389)

7 (health behaviour* or health behavior*).mp. (49894)

8 self care.mp. (35630)

9 self efficacy.mp. or Self Concept/ or Self Efficacy/ (74118)

10 self concept.mp. (52820)

11 self confidence.mp. (2590)

12 Self-Help Groups/ (8607)

13 Social Support/ or social support*.mp. (71769)

14 support group*.mp. (5366)

15 patient* participat*.mp. or Patient Participation/ (29554)

16 client* participat*.mp. (234)

17 consumer participat*.mp. or Consumer Participation/ (15873)

18 Problem Solving/ or problem solving.mp. (31319)

19 decision making.mp. or Decision Making/ (147161)

20 attitud* to health.mp. or Attitude to Health/ (181507)

21 (health attitude* or patient attitude* or client attitude*).mp. (1368)

22 Professional-Patient Relations/ or professional patient relation*.mp. (39371)

23 Physician-Patient Relations/ or physician patient relation*.mp. (67816)

24 Interpersonal Relations/ or interpersonal relation*.mp. (67381)

25 Communication/ or communication.mp. (264473)

26 Patient-Centered Care/ or patient centred.mp. (16857)

27 patient centered.mp. (19661)

28 Cooperative Behavior/ (38752)

29 cooperat*.mp. (179949)

30 collaborat*.mp. (96116)

31 partnership.mp. (17148)

32 health partner*.mp. (501)

33 consumer health information.mp. or Consumer Health Information/ (3000)

34 Health Knowledge, Attitudes, Practice/ or health knowledge.mp. (92274)

35 health information.mp. (17517)

36 health education.mp. or Health Education/ (76817)

37 Health Promotion/ or health promot*.mp. (76833)

38 patient* educat*.mp. or Patient Education as Topic/ (90169)

39 information seeking.mp. or Information Seeking Behavior/ (2598)

40 Health Literacy/ or health litera*.mp. (6982)

41 client* educat*.mp. (191)

42 self educat*.mp. (264)

43 "Patient Acceptance of Health Care"/ or help seeking.mp. (39526)

44 treatment seeking.mp. (3315)

45 health care utili?ation.mp. (5055)

46 health care seeking.mp. (702)

47 1 or 2 (27873)

48 3 or 4 or 5 or 6 or 7 or 8 or 9 or 10 or 11 or 12 or 13 or 14 or 15 or 16 or 17 or 18 or 19 or 20 or 21 or 22 or 23 or 24 or 25 or 26 or 27 or 28 or 29 or 30 or 31 or 32 or 33 or 34 or 35 or 36 or 37 or 38 or 39 or 40 or 41 or 42 or 43 or 44 or 45 or 46 (1358167)

49 47 and 48 (553)

50 limit 49 to english language (492)

***************************

**Scopus database**

(TITLE-ABS-KEY(endometrio*)) AND (((TITLE-ABS-KEY("self manag*")) OR (TITLE-ABS-KEY("self help")) OR (TITLE-ABS-KEY("expert patient*")) OR (TITLE-ABS-KEY("health behavior*")) OR (TITLE-ABS-KEY("health behaviour*")) OR (TITLE-ABS-KEY("self care")) OR (TITLE-ABS-KEY("self efficacy")) OR (TITLE-ABS-KEY("self concept"))) OR ((TITLE-ABS-KEY("self confidence")) OR (TITLE-ABS-KEY("social support*")) OR (TITLE-ABS-KEY("support group*")) OR (TITLE-ABS-KEY("patient* participat*")) OR (TITLE-ABS-KEY("client participat*")) OR (TITLE-ABS-KEY("consumer participat*")) OR (TITLE-ABS-KEY("problem solving"))) OR ((TITLE-ABS-KEY("decision making")) OR (TITLE-ABS-KEY("attitud* to health")) OR (TITLE-ABS-KEY("health attitude*" OR "patient* attitude*" OR "client* attitude*")) OR (TITLE-ABS-KEY("professional patient relation*")) OR (TITLE-ABS-KEY("physician patient relation*")) OR (TITLE-ABS-KEY("interpersonal relation*")) OR (TITLE-ABS-KEY(communication))) OR ((TITLE-ABS-KEY("patient centred" OR "patient centered")) OR (TITLE-ABS-KEY(cooperat*)) OR (TITLE-ABS-KEY(collaborat*)) OR (TITLE-ABS-KEY(partnership)) OR (TITLE-ABS-KEY("health partner*")) OR (TITLE-ABS-KEY("consumer health information")) OR (TITLE-ABS-KEY("health knowledge"))) OR ((TITLE-ABS-KEY("health information")) OR (TITLE-ABS-KEY("health education")) OR (TITLE-ABS-KEY("health promot*")) OR (TITLE-ABS-KEY("patient* educat*")) OR (TITLE-ABS-KEY("information seeking")) OR (TITLE-ABS-KEY("health litera*")) OR (TITLE-ABS-KEY("client* educat*"))) OR ((TITLE-ABS-KEY("self educat*")) OR (TITLE-ABS-KEY("help seeking")) OR (TITLE-ABS-KEY("treatment seeking")) OR (TITLE-ABS-KEY("health care utili?ation")) OR (TITLE-ABS-KEY("health care seeking")))) AND ( LIMIT-TO ( LANGUAGE,"English" ) )

**Web of science**

| **Set** | \|  \|  \| \| --- \| --- \| |
| --- | --- | --- | --- |
| #40 | (#39 AND #1) *AND***LANGUAGE:** (English)  *DocType=All document types; Language=All languages;* |
| #39 | #38 OR #37 OR #36 OR #35 OR #34 OR #33 OR #32 OR #31 OR #30 OR #29 OR #28 OR #27 OR #26 OR #25 OR #24 OR #23 OR #22 OR #21 OR #20 OR #19 OR #18 OR #17 OR #16 OR #15 OR #14 OR #13 OR #12 OR #11 OR #10 OR #9 OR #8 OR #7 OR #6 OR #5 OR #4 OR #3 OR #2  *DocType=All document types; Language=All languages;* |
| #38 | **TOPIC:** ("health care utili?ation")  *DocType=All document types; Language=All languages;* |
| #37 | TS=("help seeking" OR "treatment seeking" OR "health care seeking")  *DocType=All document types; Language=All languages;* |
| #36 | TS=("client* educat*" OR "self educat*")  *DocType=All document types; Language=All languages;* |
| #35 | **TOPIC:** ("health litera*")  *DocType=All document types; Language=All languages;* |
| #34 | **TOPIC:** ("information seeking")  *DocType=All document types; Language=All languages;* |
| #33 | **TOPIC:** ("patient* educat*")  *DocType=All document types; Language=All languages;* |
| #32 | **TOPIC:** ("health promot*")  *DocType=All document types; Language=All languages;* |
| #31 | **TOPIC:** ("health education")  *DocType=All document types; Language=All languages;* |
| #30 | **TOPIC:** ("health information")  *DocType=All document types; Language=All languages;* |
| #29 | **TOPIC:** ("health knowledge")  *DocType=All document types; Language=All languages;* |
| #28 | **TOPIC:** ("consumer health information")  *DocType=All document types; Language=All languages;* |
| #27 | **TOPIC:** ("health partner*")  *DocType=All document types; Language=All languages;* |
| #26 | **TOPIC:** (partnership)  *DocType=All document types; Language=All languages;* |
| #25 | **TOPIC:** (collaborat*)  *DocType=All document types; Language=All languages;* |
| #24 | **TOPIC:** (cooperat*)  *DocType=All document types; Language=All languages;* |
| #23 | **TOPIC:** ("patient centred" OR "patient centered")  *DocType=All document types; Language=All languages;* |
| #22 | **TOPIC:** ("communication")  *DocType=All document types; Language=All languages;* |
| #21 | **TOPIC:** ("interpersonal relation*")  *DocType=All document types; Language=All languages;* |
| #20 | **TOPIC:** ("physician patient relation*")  *DocType=All document types; Language=All languages;* |
| #19 | **TOPIC:** ("professional patient relation*")  *DocType=All document types; Language=All languages;* |
| #18 | **TOPIC:** ("health attitude*" OR "patient* attitude*" OR "client* attitude*")  *DocType=All document types; Language=All languages;* |
| #17 | **TOPIC:** ("attitud* to health")  *DocType=All document types; Language=All languages;* |
| #16 | **TOPIC:** ("decision making")  *DocType=All document types; Language=All languages;* |
| #15 | **TOPIC:** ("problem solving")  *DocType=All document types; Language=All languages;* |
| #14 | **TOPIC:** ("consumer participat*")  *DocType=All document types; Language=All languages;* |
| #13 | **TOPIC:** ("client* participat*")  *DocType=All document types; Language=All languages;* |
| #12 | **TOPIC:** ("patient* participat*")  *DocType=All document types; Language=All languages;* |
| #11 | **TOPIC:** ("support group*")  *DocType=All document types; Language=All languages;* |
| #10 | **TOPIC:** ("social support")  *DocType=All document types; Language=All languages;* |
| #9 | **TOPIC:** ("self confidence")  *DocType=All document types; Language=All languages;* |
| #8 | **TOPIC:** ("self concept")  *DocType=All document types; Language=All languages;* |
| #7 | **TOPIC:** ("self efficacy")  *DocType=All document types; Language=All languages;* |
| #6 | **TOPIC:** ("self care")  *DocType=All document types; Language=All languages;* |
| #5 | **TOPIC:** ("health behavior*" OR "health behaviour")  *DocType=All document types; Language=All languages;* |
| #4 | **TOPIC:** ("expert patient*")  *DocType=All document types; Language=All languages;* |
| #3 | **TOPIC:** ("self help")  *DocType=All document types; Language=All languages;* |
| #2 | **TOPIC:** ("self manag*")  *DocType=All document types; Language=All languages;* |
| #1 | **TOPIC:** (endometrio*) *DocType=All document types; Language=All languages;* |

**Cinahl database**

| **#** | **Query** | **Limiters/Expanders** |
| --- | --- | --- |
| S49 | S47 AND S48 | Limiters - English Language  Search modes - Boolean/Phrase |
| S48 | S3 OR S4 OR S5 OR S6 OR S7 OR S8 OR S9 OR S10 OR S11 OR S12 OR S13 OR S14 OR S15 OR S16 OR S17 OR S18 OR S19 OR S20 OR S21 OR S22 OR S23 OR S24 OR S25 OR S26 OR S27 OR S28 OR S29 OR S30 OR S31 OR S32 OR S33 OR S34 OR S35 OR S36 OR S37 OR S38 OR S39 OR S40 OR S41 OR S42 OR S43 OR S44 OR S45 OR S46 | Search modes - Boolean/Phrase |
| S47 | S1 OR S2 | Search modes - Boolean/Phrase |
| S46 | "health care seeking" | Search modes - Boolean/Phrase |
| S45 | "health care utili?ation" | Search modes - Boolean/Phrase |
| S44 | "treatment seeking" | Search modes - Boolean/Phrase |
| S43 | (MH "Help Seeking Behavior") OR "help seeking" | Search modes - Boolean/Phrase |
| S42 | "self educat*" | Search modes - Boolean/Phrase |
| S41 | "client* educat*" | Search modes - Boolean/Phrase |
| S40 | (MH "Health Literacy") OR "health litera*" | Search modes - Boolean/Phrase |
| S39 | (MH "Information Seeking Behavior") OR "information seeking" | Search modes - Boolean/Phrase |
| S38 | (MH "Patient Education") OR "patient* educat*" | Search modes - Boolean/Phrase |
| S37 | (MH "Health Promotion") OR "health promot*" | Search modes - Boolean/Phrase |
| S36 | (MH "Health Education") OR "health education" | Search modes - Boolean/Phrase |
| S35 | (MH "Health Information") OR "health information" | Search modes - Boolean/Phrase |
| S34 | (MH "Health Knowledge") OR "health knowledge" | Search modes - Boolean/Phrase |
| S33 | (MH "Consumer Health Information") OR "consumer health information" | Search modes - Boolean/Phrase |
| S32 | "health partner*" | Search modes - Boolean/Phrase |
| S31 | partnership | Search modes - Boolean/Phrase |
| S30 | (MH "Collaboration") OR collaborat* | Search modes - Boolean/Phrase |
| S29 | cooperat* | Search modes - Boolean/Phrase |
| S28 | (MH "Cooperative Behavior") | Search modes - Boolean/Phrase |
| S27 | (MH "Communication") OR communication | Search modes - Boolean/Phrase |
| S26 | (MH "Patient Centered Care") OR "patient centered" OR "patient centred" | Search modes - Boolean/Phrase |
| S25 | (MH "Interpersonal Relations") OR "interpersonal relation*" | Search modes - Boolean/Phrase |
| S24 | (MH "Professional-Patient Relations") OR (MH "Physician-Patient Relations") OR "professional patient relation*" OR "physician patient relation*" | Search modes - Boolean/Phrase |
| S23 | (MH "Consumer Attitudes") OR (MH "Patient Attitudes") OR "health attitude*" OR "patient* attitude*" OR "client* attitude*" | Search modes - Boolean/Phrase |
| S22 | (MH "Attitude to Health") OR "attitud* to health" | Search modes - Boolean/Phrase |
| S21 | (MH "Decision Making") OR "decision making" | Search modes - Boolean/Phrase |
| S20 | (MH "Problem Solving") OR "problem solving" | Search modes - Boolean/Phrase |
| S19 | (MH "Consumer Participation") OR "consumer participat*" | Search modes - Boolean/Phrase |
| S18 | "client* participat*" | Search modes - Boolean/Phrase |
| S17 | "patient* participat*" | Search modes - Boolean/Phrase |
| S16 | "support group*" | Search modes - Boolean/Phrase |
| S15 | "social support" | Search modes - Boolean/Phrase |
| S14 | (MH "Support Groups") | Search modes - Boolean/Phrase |
| S13 | "self confidence" | Search modes - Boolean/Phrase |
| S12 | "self concept" | Search modes - Boolean/Phrase |
| S11 | (MH "Self Concept") OR (MH "Self-Efficacy") | Search modes - Boolean/Phrase |
| S10 | "self efficacy" | Search modes - Boolean/Phrase |
| S9 | "self care" | Search modes - Boolean/Phrase |
| S8 | "health behavior*" OR "health behaviour*" | Search modes - Boolean/Phrase |
| S7 | (MH "Health Behavior") | Search modes - Boolean/Phrase |
| S6 | "expert patient*" | Search modes - Boolean/Phrase |
| S5 | "self help" | Search modes - Boolean/Phrase |
| S4 | "self manag*" | Search modes - Boolean/Phrase |
| S3 | (MH "Self Care") | Search modes - Boolean/Phrase |
| S2 | endometrio* | Search modes - Boolean/Phrase |
| S1 | (MH "Endometriosis") | Search modes - Boolean/Phrase |
