# Supplementary material for: Self-management in condition-specific health: a systematic review of the evidence among women diagnosed with endometriosis
Source: BMC Womens Health. 2019 Jun 19;19:80. doi: 10.1186/s12905-019-0774-6 (PMC6585070; doi:10.1186/s12905-019-0774-6)
Supplement: Supplementary file 2 — Table S1. Quality assessment scores for the papers involving quantitative studies. (DOCX 18 kb) [file 12905_2019_774_MOESM2_ESM.docx]

**Supplementary Table 1: Quality assessment scores for the papers involving quantitative studies**

| Paper | Question /objective sufficiently described | Design evident & appropriate | Subject selection described & appropriate | Subject characteristic sufficiently described | Random allocation to treatment described (if possible) | Blinding investigators reported (if possible) | Blinding participants reported (if possible) | Measures defined & robust. Means of assessment reported | Sample  size appropriate | Analysis described, & appropriate | Variance reported | Controlled for confounding | Results reported in sufficient detail | Results support conclusion | Score |
| --- | --- | --- | --- | --- | --- | --- | --- | --- | --- | --- | --- | --- | --- | --- | --- |
| **Randomised Controlled Trials** | | | | | | | | | | | | | | | |
| Mira, 2015 [17] | Yes | Yes | Yes | Yes | Yes | No | N/A | Yes | Partial | Yes | Yes | Partial | Yes | Yes | 0.85 |
| Goncalves, 2017  [18] ◼ | Yes | Yes | Yes | Yes | Yes | No | N/A | Yes | Partial | Yes | Partial | Partial | Yes | Yes | 0.81 |
| **Survey studies** | | | | | | | | | | | | | | | |
| Whitney, 1998  [24] | Partial | Partial | Partial | Partial | N/A | N/A | N/A | No | N/A | Partial | N/A | N/A | Partial | Partial | 0.44 |
| Ballweg, 2004  [19] | No | Partial | Partial | No | N/A | N/A | N/A | No | Partial | Partial | No | N/A | Partial | Partial | 0.30 |
| Music, 2005  [25] | No | No | No | No | N/A | N/A | N/A | No | N/A | Partial | No | N/A | Partial | Partial | 0.17 |
| Bodén, 2013  [20] | Yes | Partial | Partial | Partial | N/A | N/A | N/A | Partial | N/A | Partial | No | N/A | Partial | Yes | 0.56 |
| Kundu, 2015  [21] | Yes | Yes | Partial | Yes | N/A | N/A | N/A | Partial | N/A | Partial | N/A | N/A | Yes | Partial | 0.75 |
| Roos-Eysbouts, 2015 [22] | Yes | Partial | Yes | Yes | N/A | N/A | N/A | Partial | N/A | Yes | Partial | N/A | Yes | Partial | 0.78 |
| Shoebotham, 2016 [23] | Yes | Partial | Partial | Partial | N/A | N/A | N/A | Partial | N/A | Partial | N/A | N/A | Yes | Yes | 0.69 |

◼🞟⧫❖•⭘⯎ Symbols indicate data generated by the same study.
