# Supplementary material for: Self-management in condition-specific health: a systematic review of the evidence among women diagnosed with endometriosis
Source: BMC Womens Health. 2019 Jun 19;19:80. doi: 10.1186/s12905-019-0774-6 (PMC6585070; doi:10.1186/s12905-019-0774-6)
Supplement: Supplementary file 3 — Table S2. Quality assessment scores for the papers involving qualitative methods. (DOCX 20 kb) [file 12905_2019_774_MOESM3_ESM.docx]

**Supplementary Table 2 – Quality assessment scores for the papers involving qualitative methods**

| Papers | Question /objective clearly described | Design evident and appropriate to answer study question | Context for study is clear | Connection to a theoretical framework/wider body of knowledge | Sampling strategy described, relevant and justified | Data collection methods clearly described and systematic | Data analysis clearly described, complete and systematic | Use of verification procedure(s) to establish credibility of the study | Conclusion supported by the results | Reflexivity of the account | Score |
| --- | --- | --- | --- | --- | --- | --- | --- | --- | --- | --- | --- |
| Cox et al. 2003 [28] ⧫ | Partial | Yes | Partial | Partial | Partial | Partial | Partial | No | Partial | No | 0.45 |
| Cox et al. 2003 [27] ⧫ | Yes | Yes | Yes | Partial | Partial | Yes | Yes | Yes | Partial | No | 0.75 |
| Jones et al. 2004 [35] | Yes | Yes | Yes | Yes | Partial | Yes | Yes | Yes | Yes | Yes | 0.95 |
| Denny 2004 [29] ❖ | Yes | Yes | Partial | Yes | Partial | Yes | Partial | Yes | Yes | No | 0.75 |
| Denny 2004 [30] ❖ | Yes | Yes | Partial | Yes | Partial | Yes | Yes | Yes | Yes | No | 0.80 |
| Strzempko Butt & Chesla 2007 [26] | Yes | Yes | Partial | Yes | Partial | Yes | Yes | Yes | Yes | Partial | 0.85 |
| Denny & Mann 2007 [32] • | Yes | Yes | Partial | Yes | Partial | Yes | Yes | Yes | Yes | No | 0.80 |
| Manderson et al. 2008 [36] ⭘ | Partial | Yes | Yes | Yes | Partial | Partial | Yes | Yes | Yes | No | 0.75 |
| Markovic et al. 2008 [37] ⭘ | Yes | Yes | Partial | Yes | Partial | Partial | Yes | Yes | Yes | No | 0.75 |
| Denny & Mann 2008 [33] • | Yes | Yes | Partial | Yes | Partial | Partial | Yes | Yes | Yes | No | 0.75 |
| Seear 2009 [40] ⯎ | Partial | Yes | Partial | Yes | Partial | Partial | Yes | No | Yes | No | 0.60 |
| Seear 2009 [41] ⯎ | Partial | Yes | Partial | Yes | Partial | Partial | Yes | No | Partial | No | 0.55 |
| Seear 2009 [42] ⯎ | Yes | Yes | Partial | Yes | No | Partial | Yes | No | Partial | No | 0.55 |
| Denny 2009 [31] • | Yes | Yes | Partial | Yes | Partial | Yes | Yes | Yes | Yes | Yes | 0.90 |
| Moradi et al. 2014 [38] | Yes | Yes | Yes | Yes | Partial | Yes | Yes | Yes | Yes | No | 0.85 |
| Gonçalves 2016 [34] ◼ | Yes | Yes | Partial | Partial | Partial | Partial | Yes | Yes | Yes | No | 0.70 |
| Roomaney & Kagee 2016 [39] | Yes | Yes | Yes | Partial | Yes | Yes | Yes | Yes | Yes | No | 0.85 |
| Young et al. 2016 [43] | Yes | Yes | Yes | Yes | Yes | Yes | Yes | Yes | Yes | No | 0.90 |

◼🞟⧫❖•⭘⯎ Symbols indicate data generated by the same study.
